# Listeriolysin S Is a Streptolysin S-Like Virulence Factor That Targets Exclusively Prokaryotic Cells *In Vivo*

**DOI:** 10.1128/mBio.00259-17

**Published:** 2017-04-04

**Authors:** Juan J. Quereda, Marie A. Nahori, Jazmín Meza-Torres, Martin Sachse, Patricia Titos-Jiménez, Jaime Gomez-Laguna, Olivier Dussurget, Pascale Cossart, Javier Pizarro-Cerdá

**Affiliations:** a Institut Pasteur, Unité des Interactions Bactéries-Cellules, Paris, France; b Institut National de la Santé et de la Recherche Médicale, U604, Paris, France; c Institut National de la Recherche Agronomique, USC2020, Paris, France; d Institut Pasteur, Ultrapole, Paris, France; e Anatomy and Comparative Pathology Department, University of Cordoba, International Excellence Agrifood Campus CeiA3, Cordoba, Spain; f Cellule Pasteur, Université Paris Diderot, Sorbonne Paris Cité, Paris, France; University of British Columbia

**Keywords:** *Listeria*, listeriolysin S, streptolysin S, cytotoxin, epidemics, infection

## Abstract

Streptolysin S (SLS)-like virulence factors from clinically relevant Gram-positive pathogens have been proposed to behave as potent cytotoxins, playing key roles in tissue infection. Listeriolysin S (LLS) is an SLS-like hemolysin/bacteriocin present among *Listeria monocytogenes* strains responsible for human listeriosis outbreaks. As LLS cytotoxic activity has been associated with virulence, we investigated the LLS-specific contribution to host tissue infection. Surprisingly, we first show that LLS causes only weak red blood cell (RBC) hemolysis *in vitro* and neither confers resistance to phagocytic killing nor favors survival of *L. monocytogenes* within the blood cells or in the extracellular space (in the plasma). We reveal that LLS does not elicit specific immune responses, is not cytotoxic for eukaryotic cells, and does not impact cell infection by *L. monocytogenes*. Using *in vitro* cell infection systems and a murine intravenous infection model, we actually demonstrate that LLS expression is undetectable during infection of cells and murine inner organs. Importantly, upon intravenous animal inoculation, *L. monocytogenes* is found in the gastrointestinal system, and only in this environment LLS expression is detected *in vivo*. Finally, we confirm that LLS production is associated with destruction of target bacteria. Our results demonstrate therefore that LLS does not contribute to *L. monocytogenes* tissue injury and virulence in inner host organs as previously reported. Moreover, we describe that LlsB, a putative posttranslational modification enzyme encoded in the LLS operon, is necessary for murine inner organ colonization. Overall, we demonstrate that LLS is the first SLS-like virulence factor targeting exclusively prokaryotic cells during *in vivo* infections.

## IMPORTANCE

The most severe human listeriosis outbreaks are caused by *L. monocytogenes* strains harboring listeriolysin S (LLS), previously described as a cytotoxin that plays a critical role in host inner tissue infection. Cytotoxic activities have been proposed as a general mode of action for streptolysin S (SLS)-like toxins, including clostridiolysin S and LLS. We now challenge this dogma by demonstrating that LLS does not contribute to virulence *in vivo* once the intestinal barrier has been crossed. Importantly, we show that intravenous *L. monocytogenes* inoculation leads to bacterial translocation to the gastrointestinal system, where LLS is specifically expressed, targeting the host gut microbiota. Our study highlights the heterogeneous modes of action of SLS-like toxins, and we demonstrate for the first time a further level of complexity for SLS-like biosynthetic clusters as we reveal that the putative posttranslational modification enzyme LlsB is actually required for inner organ colonization, independently of the LLS activity.

## INTRODUCTION

*Listeria monocytogenes* is a foodborne pathogen and a facultative intracellular bacterium capable of causing severe disease in humans and animals. Upon ingestion of contaminated food, *L. monocytogenes* colonizes the intestine and crosses the intestinal barrier, disseminating via the blood to the liver, spleen, brain, and placenta (1, 2). The listeriosis fatality rate is estimated to be 20 to 30% of infected individuals despite antibiotic treatment (1). The most severe human listeriosis outbreaks are associated with a subset of *L. monocytogenes* lineage I strains that harbor a gene cluster encoding the bacteriocin and hemolytic factor listeriolysin S (LLS) (3, 4). Interestingly, the LLS gene cluster is absent from the most commonly studied *L. monocytogenes* lineage II strains EGD, EGDe, and 10403S (3), and its contribution to the intracellular lifecycle of *L. monocytogenes* is unknown (5).

LLS is homologous to streptolysin S (SLS [encoded by *sagA* in the *sag* operon]), a potent cytolytic toxin produced by most group A *Streptococcus pyogenes* (GAS) strains (6, 7). SLS is naturally expressed *in vitro* and is responsible for the classical β-hemolytic phenotype of *S. pyogenes* on blood agar plates (8). Using live-cell imaging, it has been shown that SLS activates the major erythrocyte anion exchange protein band 3 and favors a rapid influx of Cl^−^ ions into red blood cells (RBCs), leading to cellular rupture (9). In HEK-293 cells, SLS causes lactate dehydrogenase (LDH) release into the medium, massive cytoskeletal disassembly, loss of focal contacts, and detachment from the tissue culture plates (7). SLS promotes resistance to phagocytic killing in whole-blood killing assays and activates an inflammatory programed cell death pathway in macrophages (10, 11). *In vivo* studies have shown that SLS is required for *S. pyogenes* infection of skin and soft tissues (9, 12). The toxin encoded by an SLS-like gene cluster in *Clostridium botulinum* is named clostridiolysin S (CLS), and similarly to SLS, it is hemolytic and cytotoxic in HEK-293 cells (7, 13).

LLS, CLS, and SLS belong to the family of thiazole/oxazole-modified microcins (TOMMs) and are encoded by biosynthetic gene clusters characterized by the presence of cyclodehydratases/dehydrogenase genes (7). In the case of the LLS gene cluster, the genes *llsB*, *llsY*, and *llsD* code for putative posttranslational modification (PTM) enzymes that modify the product of the *llsA* gene coding for the structural LLS toxin (6, 7). Attempts to uncover the structural characteristics of the mature SLS-like toxins have been unsuccessful (7, 12, 13). The only direct structural insight available suggests that SLS contains two oxazole moieties observed at positions Ser^46^ and Ser^48^ and that CLS contains a methyloxazole at position Thr^46^ (13).

It has been suggested that given the genetic similarities of the TOMM operons in Gram-positive pathogens, it is likely that these TOMMs contribute to the pathogenic potential of each bacterial producer, similarly to SLS for *S. pyogenes* (6, 14). However, it is currently unknown if all SLS-like molecules of bacterial pathogens are cytotoxins, which play a role in tissue injury and contribute to virulence by targeting eukaryotic cells. By using an *llsB* deletion mutant, it has been previously concluded that LLS is a hemolysin and a cytotoxin that contributes to *L. monocytogenes* virulence in an intraperitoneal murine model of infection (3). We recently described that in orally infected mice, LLS behaves as a bacteriocin favoring *L. monocytogenes* gut colonization through modulation of the intestinal microbiota (4).

In the present study, we sought to deepen our insight into the functions of LLS during listeriosis, with a particular focus on its effects once the intestinal barrier has been crossed. Here, we show that while LLS kills prokaryotic targets, it does not impact infection of eukaryotic host cells *in vitro* and *in vivo*. Our present results demonstrate that, apart from its role as a bacteriocin in the host intestine, the structural product of the *llsA* gene has negligible activity on eukaryotic host cells, while the *llsB* gene product contributes to colonization of deep organs.

## RESULTS

### LLS causes weak RBC hemolysis *in vitro* and does not alter RBC counts *in vivo.*

**FIG 1:**
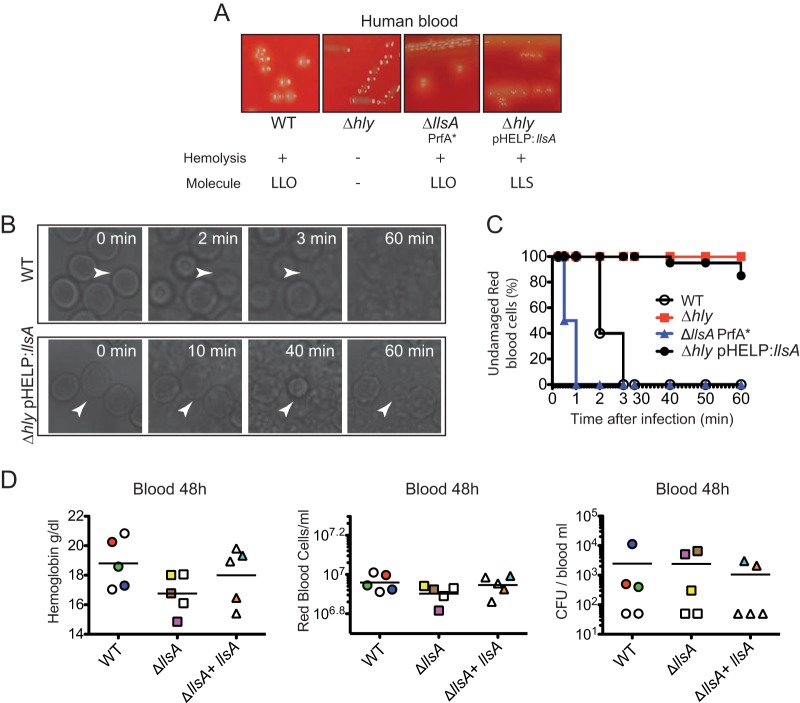
Hemolytic and cytolytic properties of LLS. (A) Hemolysis of human blood BHI agar plates by *L. monocytogenes* F2365, *L. monocytogenes* F2365 Δ*hly*, *L. monocytogenes* F2365 Δ*hly*(pHELP::*llsA*), and *L. monocytogenes* Δ*llsA* PrfA*. The lower panel shows the presence (+) or absence (−) of hemolysis and the *L. monocytogenes* molecule responsible for such hemolysis. LLO, listeriolysin O; LLS, listeriolysin S. (B and C) Live microscopy of RBC hemolysis using phase-contrast microscopy at different times posttreatment with the same strains used in panel A. Panel C shows quantification of the hemolysis over time. Data from one representative experiment out of the three performed are shown. (D) Concentration of hemoglobin (grams per deciliter), number of RBCs per milliliter of blood, and number of *L. monocytogenes* CFU per milliliter of blood of BALB/c mice injected intravenously with 10^4^ CFU of the indicated strains. Mice were killed at 48 h p.i., and a blood sample was taken to assess the number of RBCs and the hemoglobin concentration. Mice whose blood contained more than 300  *L. monocytogenes* CFU/ml are colored to facilitate comparison of blood parameters.

By growing *L. monocytogenes* colonies that constitutively express LLS in blood agar plates, or by using cell-free supernatants obtained from bacteria activated by the LLS inducer yeast RNA core, LLS has been previously shown to display hemolytic activity (3). In the case of SLS, real-time imaging indicated that the secreted toxin induces hemolysis of RBCs in 30 s (9). We investigated whether LLS exerts hemolysis as efficiently as SLS. First, we determined in brain heart infusion (BHI) agar plates with human or mouse blood the hemolytic activity of the *L. monocytogenes* F2365 wild type (WT), F2365 Δ*hly*, F2365 Δ*llsA* PrfA* (a strain that constitutively expresses listeriolysin O [LLO]), and F2365 Δ*hly*(pHELP::*llsA*) (a strain that does not produce LLO but expresses LLS). F2365 Δ*hly* was not hemolytic on human (Fig. 1A) or mouse (see Fig. S1 in the supplemental material) BHI blood agar plates. On the other hand, the F2365 WT and Δ*llsA* PrfA* strains caused hemolysis of both RBCs due to LLO activity, while the Δ*hly*(pHELP::*llsA*) strain also caused hemolysis in human and mouse RBCs due to LLS activity (Fig. 1A; Fig. S1). Second, we performed live-cell microscopy to visualize human RBC hemolysis caused by the same F2365 strains. Real-time imaging indicated 100% RBC hemolysis in 3 min due to LLO activity when incubated with the F2365 WT (Fig. 1B, upper panel, and C) or in 1 min when incubated with F2365 PrfA* Δ*llsA* (Fig. 1C). Hemolysis was marked by the visual rupture of the RBC membranes, as previously described for SLS (9). F2365 Δ*hly* caused no hemolysis, highlighting the potent activity of LLO (Fig. 1C). Surprisingly, only 15% of RBCs were lysed after 60 min of incubation with F2365 Δ*hly*(pHELP::*llsA*) (Fig. 1B, lower panel, and C), suggesting that the hemolytic activity of LLS is much less efficient than that of LLO, as shown in our experiments and as reported in the literature for SLS.

10.1128/mBio.00259-17.1 FIG S1  Hemolytic properties of LLS. (A) Hemolysis of mouse blood BHI agar plates by *L. monocytogenes* F2365, *L. monocytogenes* F2365 Δ*hly*, *L. monocytogenes* F2365 Δ*hly*(pHELP::*llsA*), and *L. monocytogenes* Δ*llsA* PrfA*. The lower panel show the presence (+) or absence (−) of hemolysis and the *L. monocytogenes* molecule responsible for such hemolysis. LLO, listeriolysin O; LLS, listeriolysin S. Download FIG S1, EPS file, 0.5 MB. Copyright © 2017 Quereda et al. 2017 Quereda et al. This content is distributed under the terms of the Creative Commons Attribution 4.0 International license.

In order to reveal the potential effects of the LLS hemolytic activity during *L. monocytogenes* infection* in vivo*, we infected intravenously conventional BALB/c mice with the F2365 WT, Δ*llsA* mutant strain, or its complemented strain and quantified the concentration of hemoglobin in blood, the number of RBCs, and the bacterial burden in blood at 48 h postinfection (p.i.). Consistent with real-time imaging performed *in vitro*, no significant changes in hemoglobin concentration (Fig. 1D, left panel) or RBC counts (Fig. 1D, center panel) were observed in mice infected with these three strains. Importantly, no correlation was observed between the number of *L. monocytogenes* cells in the blood (Fig. 1D, right panel) and the number of RBCs or hemoglobin (Fig. 1D; see Table S1 in the supplemental material). Taken together, these results show that LLS hemolytic activity is weak *in vitro* and that this activity does not affect RBC numbers during *L. monocytogenes* infection *in vivo*.

10.1128/mBio.00259-17.5 TABLE S1  Hemoglobin concentration, RBC counts, and CFU in infected mice. BALB/c mice were injected intravenously with 10^4^ CFU of the indicated strains. Mice were killed at 48 h p.i., and a blood sample was obtained to assess bacterial loads, RBC numbers, and hemoglobin concentrations. Download TABLE S1, DOC file, 0.1 MB. Copyright © 2017 Quereda et al. 2017 Quereda et al. This content is distributed under the terms of the Creative Commons Attribution 4.0 International license.

### LLS expression does not confer resistance to phagocytic clearance.

**FIG 2:**
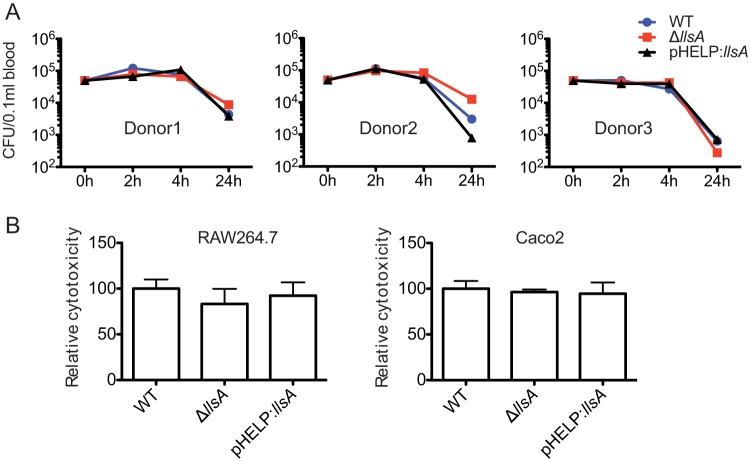
LLS expression does not confer resistance to phagocytic clearance and is not cytotoxic for eukaryotic cells. (A) Survival in human whole blood of the *L. monocytogenes* F2365 WT, *L. monocytogenes* F2365 Δ*llsA*, and *L. monocytogenes*(pHELP::*llsA*) strains. CFU numbers were monitored at 2, 4, and 24 h p.i. Experiments with three independent blood donors with 6 replicates in each experiment were performed. Error bars represent standard deviation (SD). (B) Cytotoxicity (LDH release) relative to F2365 WT (100%) in RAW264.7 and Caco-2 cells infected for 24 h (washed after 1 h of infection and with 40 µg/ml gentamicin added). Error bars represent SD.

The weak hemolytic activity of LLS prompted us to evaluate its contribution to *L. monocytogenes* survival in blood. In the case of GAS, WT strains are able to proliferate in human blood, while SLS-negative mutants are cleared, demonstrating a crucial role for SLS in the resistance to phagocytic killing in human blood (10). To explore whether LLS contributes to phagocytic clearance, we incubated fresh human blood from three donors with *L. monocytogenes* F2365 WT, F2365 Δ*llsA*, and F2365(pHELP::*llsA*) strains. No significant difference could be observed between the three strains in phagocytic clearance (Fig. 2A). GAS can kill macrophages through the activation of an inflammatory programed cell death pathway mediated by SLS and streptolysin O (SLO) (11). Moreover, SLS induces alterations in keratinocyte inflammatory signaling cascades during GAS infection (15). These SLS properties prompted us to investigate a hypothetical role of LLS in cytokine secretion by macrophages infected by *L. monocytogenes*. We used an array kit detecting 111 different cytokines (Proteome Profiler mouse XL cytokine array) to compare the inflammatory responses of RAW264.7 macrophages infected with the F2365 WT strain (which does not express LLS *in vitro*, as shown above) to the responses of cells infected with the Δ*llsA* deletion mutant or with the pHELP::*llsA* strain (which constitutively expresses LLS). No significant difference between the three groups (WT, Δ*llsA*, and pHELP::*llsA* strains) could be observed for secreted cytokine levels, including interleukin-1 (IL-1), IL-6, and tumor necrosis factor alpha (TNF-α), whose role is essential in the response to *L. monocytogenes* infection (see Fig. S2 in the supplemental material) (16), suggesting that LLS does not alter cytokine production in macrophages. Overall, our results indicate that LLS does not contribute to *L. monocytogenes* survival during the blood stage.

10.1128/mBio.00259-17.2 FIG S2  Cytokine production of RAW264.7 cells upon infection with *L. monocytogenes* F2365, *L. monocytogenes* F2365 Δ*llsA*, and *L. monocytogenes*(pHELP::*llsA*). The cytokine responses of macrophages infected during 24 h at an MOI of 2 with *L. monocytogenes* F2365 WT were compared to the responses of those infected with the Δ*llsA* deletion mutant or with the overexpressing strain *L. monocytogenes* F2365(pHELP::*llsA*) by using an array kit detecting 111 different cytokines (Proteome Profiler mouse XL cytokine array). Noninternalized bacteria were removed 1 h after infection by washing and adding gentamicin at 40 µg/ml. Twenty-four hours following infection, the cell supernatants were filtered and analyzed with the cytokine array. Download FIG S2, EPS file, 3 MB. Copyright © 2017 Quereda et al. 2017 Quereda et al. This content is distributed under the terms of the Creative Commons Attribution 4.0 International license.

### LLS expressed by intracellular *L. monocytogenes* is cytotoxic for neither macrophages nor epithelial cells.

The absence of a role for LLS in macrophages contrasts with the previously published role of this molecule as cytotoxic for diverse cell lines, including professional phagocytes (3). To revisit this concept, we evaluated LLS cytotoxicity by measuring lactate dehydrogenase (LDH) release from RAW264.7 macrophages and enterocyte-like Caco-2 cells. No differences in LDH release into culture supernatants were observed between the WT, Δ*llsA*, and pHELP::*llsA* strains in RAW264.7 and Caco-2 cells (Fig. 2B). Taken together, these results suggest that LLS secreted by intracellular *L. monocytogenes* is not cytotoxic for eukaryotic host cells.

### *llsA* promoter activity is undetectable during infection of eukaryotic cells *in vitro* or infection of deep organs, such as spleen or liver, *in vivo.*

**FIG 3:**
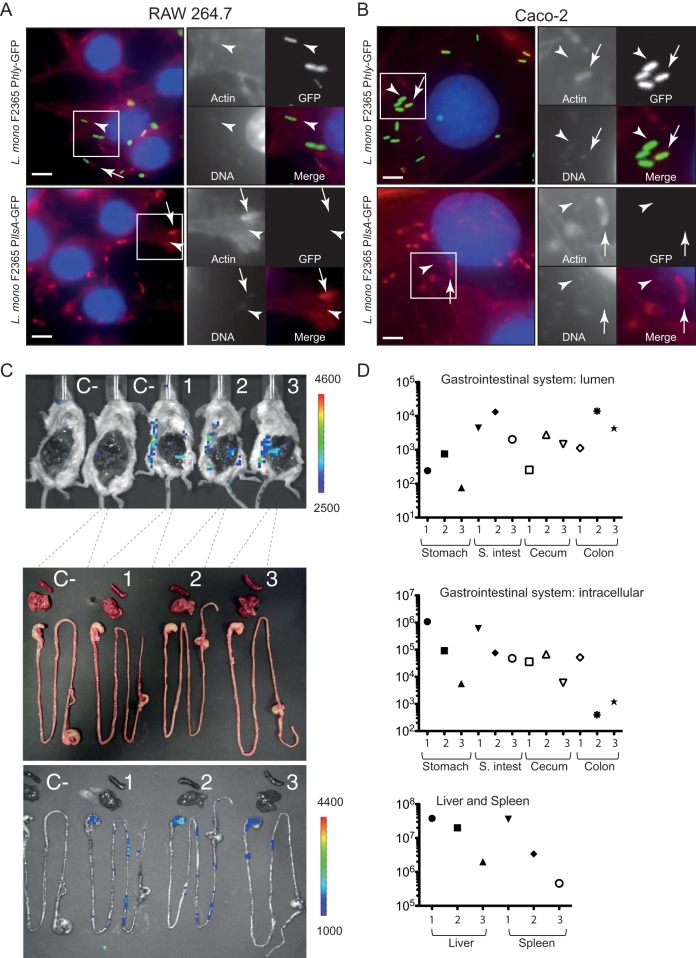
Fluorescence microscopy and bioluminescence assays to evaluate the promoter activity of llsA. RAW264.7 cells (A**)** and Caco-2 cells (B) were cultured in 96-well plates and infected with *L. monocytogenes* F2365^InlB^(pAD-P*llsA*-GFP) or *L. monocytogenes* F2365^InlB^(pAD-P*hly*-GFP). Host cells were infected for 6 h (washed after 1 h of infection and with 40 µg/ml gentamicin to kill extracellular bacteria) and fixed. GFP is shown in green. Actin (red) and nuclei (blue) were labeled with phalloidin conjugated to Alexa 546 and Hoechst, respectively. Scale bars, 5 µm. (C) Bioluminescence imaging showing induction of the LLS promoter in the gastrointestinal system after intravenous inoculation of three mice with 10^4^ bacteria per BALB/c mouse. C−, noninfected control mice. Images were acquired at 96 h p.i. with an IVIS Spectrum imaging system. The false color bar indicates the number of photons/second. (D) Bacterial counts in the stomach, small intestine (S. intest), cecum, and colon content (top), as well as in the stomach, small intestine, cecum, colon, liver, and spleen tissues (middle and bottom), of the same mice at 96 h p.i.

Transcriptional fusion of the LLS promoter to a *lux* reporter plasmid previously demonstrated that LLS expression is specifically triggered in the intestine of orally infected mice and undetectable in other organs, such as liver and spleen (although these organs contained higher bacterial counts) (4). Bioluminescent signals in the intestine of germfree mice could also be detected, indicating that the intestine itself and not the gut microbiota activates the LLS promoter (4). Different compounds naturally present in the intestine, like mucin, gastric fluid, pepsin, NaHCO_3_, short-chain fatty acids, ethanolamine, or even 6% O_2_ or intestinal content of mice added *ex vivo* could not induce bioluminescence under *in vitro* conditions (4). In the present study, we investigated whether macrophages or epithelial cells could trigger LLS expression. We generated a transcriptional fusion of the LLS promoter to the promoterless green fluorescent protein gene (*gfp*), which is more sensitive as a reporter than the luciferase systems in other Gram-positive bacteria (17). A similar green fluorescent protein (GFP) transcriptional reporter was used for *hly*, a gene essential for the intracellular lifestyle of *L. monocytogenes*. We next infected Caco-2 epithelial cells and RAW264.7 macrophages with F2365^InlB^(pAD-P*llsA*-GFP) or F2365^InlB^(pAD-P*hly*-GFP) for 2, 6, and 24 h. Figure 3 shows intracellular bacteria that polymerize host cell actin (arrows) and bacteria not associated with actin (arrowheads), presumably inside vacuoles after 5 h of gentamicin treatment. Intracellular F2365^InlB^(pAD-P*hly*-GFP) produced a green fluorescence at 2, 6, and 24 h p.i. in both cell lines tested, indicating that the GFP reporter is functional and that the *hly* promoter is active (Fig. 3A and B; see Fig. S3 and S4 in the supplemental material). Interestingly, the activity of the *llsA* promoter was undetectable at 2, 6, or 24 h p.i. in the cell lines tested (Fig. 3A and B; Fig. S3 and S4). No signal was observed for the *llsA* promoter at 2, 6, and 24 h p.i. in other cell lines (LoVo, HeLa, and Jeg-3) where the *hly* promoter was active (data not shown).

10.1128/mBio.00259-17.3 FIG S3  Fluorescence microscopy to evaluate the promoter activity of *llsA* and *hly*. RAW264.7 cells (A) and Caco-2 cells (B) were cultured in 96-well plates and infected with *L. monocytogenes* F2365^InlB^(pAD-P*llsA*-GFP) or *L. monocytogenes* F2365^InlB^(pAD-P*hly*-GFP). Host cells were infected for 2 h (washed after 1 h of infection and with 40 µg/ml gentamicin added to kill extracellular bacteria) and fixed. GFP is shown in green. Actin (red) and nuclei (blue) were labeled with phalloidin conjugated to Alexa 546 and Hoechst, respectively. Scale bars, 5 µm. Download FIG S3, EPS file, 5.2 MB. Copyright © 2017 Quereda et al. 2017 Quereda et al. This content is distributed under the terms of the Creative Commons Attribution 4.0 International license.

10.1128/mBio.00259-17.4 FIG S4  Fluorescence microscopy to evaluate the promoter activity of *llsA* and *hly*. RAW264.7 cells (A) and Caco-2 cells (B) were cultured in 96-well plates and infected with *L. monocytogenes* F2365^InlB^(pAD-P*llsA*-GFP) or *L. monocytogenes* F2365^InlB^(pAD-P*hly*-GFP). Host cells were infected for 24 h (washed after 1 h of infection and with 40 µg/ml gentamicin added to kill extracellular bacteria) and fixed. GFP is shown in green. Actin (red) and nuclei (blue) were labeled with phalloidin conjugated to Alexa 546 and Hoechst, respectively. Scale bars, 5 µm. Download FIG S4, EPS file, 5.3 MB. Copyright © 2017 Quereda et al. 2017 Quereda et al. This content is distributed under the terms of the Creative Commons Attribution 4.0 International license.

Furthermore, we investigated whether *L. monocytogenes* inoculated intravenously expresses LLS in deep organs, such as the spleen or the liver. We used the *L. monocytogenes* F2365^*llsA*::*lux*^ strain in which we fused the LLS promoter to the *lux* reporter plasmid (4). Upon intravenous infection of BALB/c mice with 10^4^ cells of F2365^*llsA*::*lux*^, a bioluminescent signal was detected in the abdomen of infected animals at 72 and 96 h p.i. To uncover the origin of the bioluminescent signals, abdominal skin and peritoneum dissection was performed. The *ex vivo* images of the gastrointestinal system and the livers and spleens removed from the body are shown in Fig. 3C. Interestingly, bioluminescent signals were only detected in the stomach and the intestine of infected mice, while being absent from the liver and spleen (which are the main organs targeted by *L. monocytogenes*). Importantly, although the bioluminescent signals were specifically expressed in the gastrointestinal system, the liver and spleen contained at least more than 100-fold CFU than the stomach, small intestine, cecum, or colon (Fig. 3D). Altogether, these results demonstrate that the *llsA* promoter is not active or is expressed at very low levels during infection of host cells, suggesting that the activation of LLS expression in the gastrointestinal system is not triggered by the infection of eukaryotic cells.

### LLS does not contribute to cell infection and is not sufficient for *L. monocytogenes* vacuolar escape.

**FIG 4:**
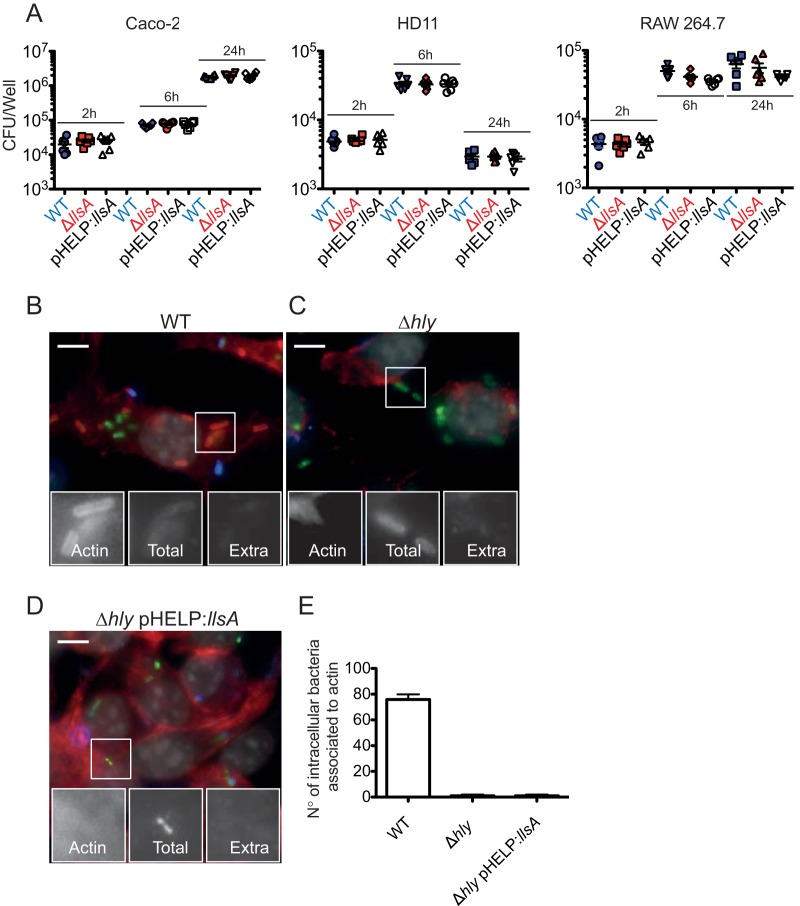
LLS expression does not confer an advantage during cell infection and is not sufficient to damage and rupture host cell vacuoles to access the host cytoplasm. (A) Numbers of viable intracellular *L. monocytogenes* F2365, *L. monocytogenes* F2365 Δ*llsA*, and *L. monocytogenes*(pHELP::*llsA*) cells in Caco-2, HD11, and RAW264.7 cells. The mean and standard error of the mean (SEM) are shown. CFU numbers were monitored at 2, 6, and 24 h p.i. (washed after 1 h of infection and with 40 µg/ml gentamicin added). Three independent experiments with 6 replicates in each experiment were performed. One representative experiment is shown. (B, C, and D) RAW264.7 cells were seeded into 96-well plates and infected with the *L. monocytogenes* F2365 WT (B), *L. monocytogenes* F2365 Δ*hly* (C), and *L. monocytogenes* F2365 Δ*hly*(pHELP::*llsA*) (D) strains. Differential immunofluorescence staining for identification of extracellular versus total *L. monocytogenes* numbers was performed. Extracellular bacteria (Extra) were labeled with a secondary goat anti-rabbit Alexa 647 (blue), and total bacteria (total) were labeled with a secondary goat anti-rabbit Alexa 488 (green) after cell permeabilization. Actin was labeled by using phalloidin conjugated to Alexa 546 (red). Nuclei were stained with Hoechst (gray). Bars, 5 µm. (E) Quantification of the number of bacteria of the *L. monocytogenes* strains used in panels B, C, and D that access the host cytosol.

Since we could not exclude that LLS produced during intestinal infection could impact the capacity of bacteria to infect eukaryotic cells, we investigated the potential contribution of LLS to infection. No differences in intracellular CFU counts were observed between the F2365 WT, F2365 Δ*llsA*, and F2365(pHELP::*llsA*) strains at 2, 6, or 24 h p.i. in RAW264.7, HD11, and Caco-2 cells (Fig. 4A). These results show that LLS is not required during cellular infection by *L. monocytogenes*.

If LLS does not play a role in cell infection, this means that it should not affect vacuolar escape by *L. monocytogenes*. The capacity of LLS to mediate vacuolar rupture and to allow intracellular growth was evaluated in RAW264.7 macrophages. Extracellular and total *L. monocytogenes* numbers were distinguished by using inside-outside staining of fluorescently labeled bacteria, and cytosolic microorganisms were identified by actin staining. Approximately 80% of F2365 WT cells escaped from vacuoles and polymerized actin (Fig. 4B and E). As expected, LLO was required for vacuolar escape in macrophages, as no escape events were observed upon cell infection with F2365 Δ*hly* (Fig. 4C and E). Importantly, no F2365 Δ*hly*(pHELP::*llsA*) cells were found associated with actin (Fig. 4D and E), suggesting that these bacteria were trapped in phagosomes and that LLS expression is not sufficient to rupture the bacterial internalization vacuole in order to facilitate access to the host cytoplasm. Overall, our results indicate that LLS does not target eukaryotic membranes during infection.

### LlsB, but not LLS, has a role in spleen and liver colonization.

**FIG 5:**
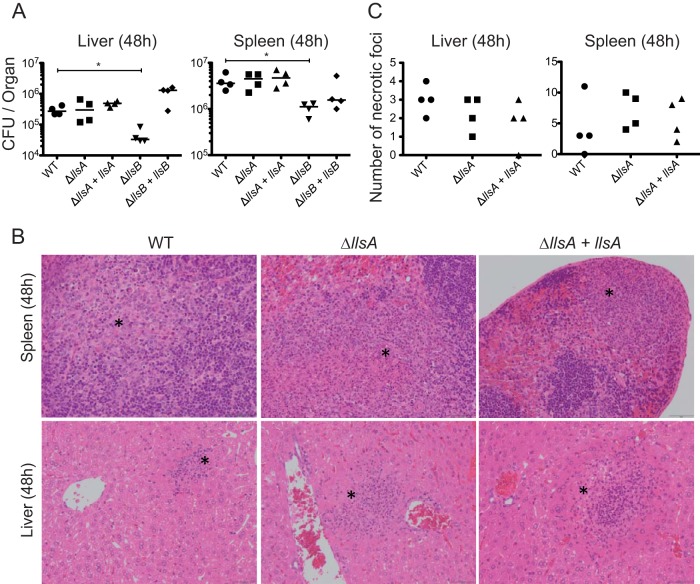
LLS role in spleen and liver colonization once the intestinal barrier has been crossed. (A) BALB/c mice were injected intravenously with 10^4^ CFU of the indicated strains. Mice were killed at 48 h p.i., and spleens and livers were removed to assess the bacterial load per organ. (Note that these numbers of CFU correspond to the half-organ used to assess bacterial load. For details, see the Materials and Methods section.) (B) Examples of spleen and liver tissues from the same infected mice. Asterisks show necrotic foci. (C) The number of necrotic foci in spleen and liver tissues from the same infected mice was evaluated. (Note that these numbers of necrotic foci correspond to the half-organ used for histopathology. For details, see the Materials and Methods section.)

SLS causes extensive tissue disruption, inflammation, and necrosis of skin lesions and is required for *S. pyogenes* infection of skin and soft-tissues (9, 10, 12). Regarding LLS, as mentioned above we have shown that this molecule is specifically expressed in the intestine of intravenously and orally infected mice, where it alters the host intestinal microbiota and increases *L. monocytogenes* persistence (Fig. 3C and 4). LLS function in the intestine requires the activity of the LlsB enzyme, which is by homology with SLS putatively involved in the posttranslational modification of LLS. As reported, deletion of LlsB completely inactivates LLS hemolytic activity *in vitro* and decreases *L. monocytogenes* virulence in a mouse intraperitoneal infection model (3). To determine the impact of LLS on virulence once *L. monocytogenes* has crossed the intestine, we intravenously infected mice with the F2365 WT, F2365 Δ*llsA*, F2365 Δ*llsB*, F2365 Δ*llsA*, and F2365 Δ*llsB* complemented strains, and quantified the bacterial burdens in the liver and spleen. In agreement with the inactivity of LLS promoter in liver and spleen (4), and with the absence of a role for LLS in the blood and in the cell lines used in the present study, the Δ*llsA* mutant strain did not display significantly different bacterial loads in the spleen or liver compared with the WT strain (Fig. 5A). However, significant differences were observed between the WT and Δ*llsB* strains in the number of CFU isolated from these organs (*P* < 0.05) (Fig. 5A). Furthermore, histopathologic assessment of the liver and spleen of mice infected with WT, Δ*llsA* mutant, and Δ*llsA* complemented strains showed no differences in the numbers, locations, and inflammatory cell types of necrotic foci (Fig. 5B and C). Together, these results demonstrate that LLS does not play an essential role in *L. monocytogenes* deep organ colonization once the intestine has been crossed and strongly suggest that LlsB performs additional important functions for virulence apart from putatively driving LLS posttranslational modification.

### LLS promotes killing of target bacteria.

**FIG 6:**
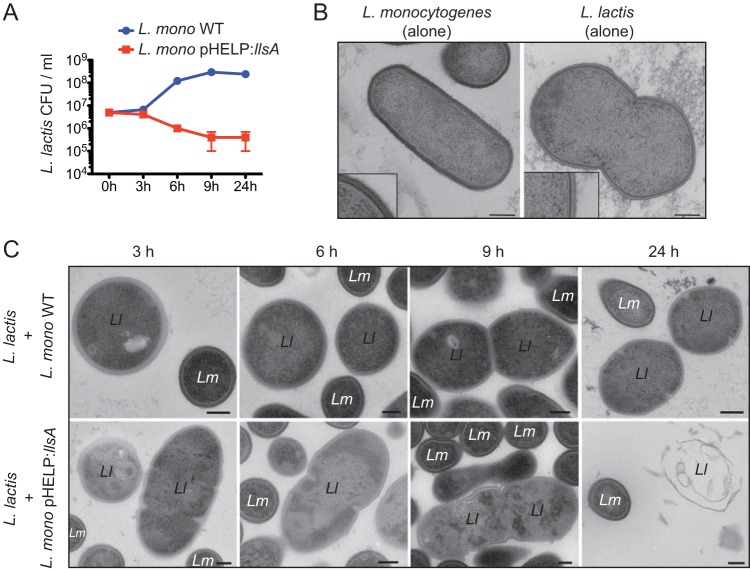
Effect of LLS on *L. lactis*. (A) Viable *L. lactis* at 3, 6, 9, and 24 h postinoculation in coculture with *L. monocytogenes* F2365 WT or *L. monocytogenes* F2365(pHELP::*llsA*). Error bars show SD. Data from one representative experiment out of the three performed are shown. (B) *L. lactis* and *L. monocytogenes* F2365 WT seen by electron microscopy when cultured alone. Bacteria were grown in BHI for 24 h. Insets present an enlargement of an area of the cell wall. Scale bars, 200 μm. (C) *L. lactis* cells cocultured with *L. monocytogenes* F2365 WT (upper panels) or *L. monocytogenes*(pHELP::*llsA*) (lower panels) in BHI for 3, 6, 9, or 24 h. Scale bars, 200 nm.

Our results suggest that during *in vivo* infections, LLS does not target eukaryotic cells. Our previous results (4) indicate that LLS is able to modulate the growth of *Lactococcus lactis*, *Staphylococcus aureus*, and *L. monocytogenes* lineage II strains as well as representatives of the *Allobaculum* and *Alloprevotella* genera during *in vivo* infection (4). To investigate a direct cytotoxic effect of LLS on target bacteria, diluted overnight cultures of *L. monocytogenes* F2365 strains (WT and the pHELP::*llsA* strain) and the target *L. lactis* were grown in coculture. After 3 h of coculture, there was a reduction in the growth of *L. lactis* only when cocultured with *L. monocytogenes* F2365(pHELP::*llsA*) (Fig. 6A). The effect of LLS on *L. lactis* growth was even higher at 6 and 9 h of coculture (Fig. 6A). Under transmission electron microscopy, *L. lactis* cultured alone or cocultured with the *L. monocytogenes* F2365 WT (which does not produce LLS under *in vitro* conditions [4]) for 3, 6, or 9 h showed the typical structure of Gram-positive cocci, with a thick, uniform, smooth cell wall and intact cytoplasmic membrane attached to the wall (Fig. 6B and C). The cytoplasm was granular and evenly distributed in the cell. Some of the cells demonstrated a dividing septum, indicative of bacterial growth (Fig. 6B and C). After 3 h of coculture with *L. monocytogenes* F2365(pHELP::*llsA*), disruption of *L. lactis* cell wall integrity was observed (Fig. 6C). At 6, 9, and 24 h of coculture with *L. monocytogenes* F2365(pHELP::*llsA*), increasing numbers of *L. lactis* cells showed more drastic changes, including cell wall wrinkles and even cellular lysis (Fig. 6C). These results confirm that LLS is the first SLS-like virulence factor of a bacterial pathogen able to promote death of target bacteria.

## DISCUSSION

In 2008, SLS-like gene clusters were discovered in clinically relevant Gram-positive pathogens (including *S. aureus* and *C. botulinum*) and other nonpathogenic bacteria (7), leading to the identification of the LLS gene cluster in *L. monocytogenes*. SLS is a potent membrane-damaging agent and a major virulence factor contributing to GAS infection through rapid destruction of eukaryotic cells and tissue damage (6, 9–11, 15, 18–20). It has been proposed that SLS-like virulence factors from other Gram-positive pathogens also behave as potent cytotoxins (6, 14). Interestingly, functional experimental data of LLS activity on eukaryotic cells are scarce (3), despite the fact that LLS is almost exclusively detected within lineage I strains (the most frequent lineage among *L. monocytogenes* clinical isolates) and that it has been related to the *L. monocytogenes* infectious potential in epidemiological and comparative genomic studies (21, 22). In the present work, we aimed to characterize the extent to which LLS potentially contributes to host infection by directly performing damaging activities on eukaryotic cells and tissues.

Using an array of molecular, cell biology, and histology techniques, we demonstrate that unlike SLS, (i) LLS causes very weak RBC hemolysis, (ii) it does not confer resistance to phagocytic clearance, (iii) it does not affect the levels of secreted cytokines by cells infected by *L. monocytogenes*, (iv) when expressed under the control of its native promoter or expressed through a constitutive promoter by intracellular *L. monocytogenes*, it is not cytotoxic for epithelial cells and macrophages, (v) its constitutive expression by *L. monocytogenes* in the confined space of a phagocytic vacuole is not sufficient to rupture this membrane compartment, (vi) its expression is undetectable within host cells due to inactivity of its promoter under both *in vitro* and *in vivo* conditions, (vii) it does not contribute to eukaryotic cell infection, and (viii) it does not contribute to virulence in an intravenous infection murine model. Altogether, our results clearly demonstrate that the biological activity of LLS is distinct from that of SLS or CLS, showing that LLS does not target eukaryotic host cells and is not involved in inner organ infection during systemic stages of listeriosis.

It has been previously proposed that LLS is hemolytic on blood agar plates and cytotoxic for epithelial and phagocytic cell lines (3). Importantly, experimental conditions *in vitro* used a ratio of 100 bacteria per cell for 6 h (without explicit use of gentamicin) to demonstrate the cytotoxic effect of LLS on J774, C2-Bbe, and CT26 cells (3). The fact that we did not observe the same cytotoxic effect on Caco-2 or RAW264.7 cells after the same infection period (making use of gentamicin after 1 h p.i.), using an MOI of 5 for Caco-2 cells or an MOI of 2 for RAW264.7 cells, leads us to believe that experimental conditions used in the previous study (3) may have influenced the *in vitro* cell system conditions, finally leading to an increase of ≈20 to 30% of LDH release to the medium due to LLS and also bacterial exposure. Moreover, the cytotoxic effect was only demonstrated by using a *L. monocytogenes* strain in which LLS was constitutively expressed using the pHELP promoter (3).

It has also been claimed that LLS contributes to virulence in a murine intraperitoneal model of infection (3). This conclusion was based on the reduced F2365 Δ*llsB* CFU numbers in the livers and spleens relative to those in the corresponding F2365 WT-infected mice. However, when we compared the virulence of the F2365 Δ*llsA* and F2365 Δ*llsB* mutants to that of the F2365 WT strain, we discovered an unexpected difference between these two deletion mutants: *llsB* but not *llsA* (the gene coding for the toxin LLS) contributed to virulence in our mouse intravenous model of infection, indicating that LlsB performs additional functions apart from the putative posttranslational modification of LLS. *llsB* is therefore the first gene from a dehydratase/dehydrogenase TOMM complex reported to perform additional functions apart from putative posttranslational modifications of the cluster-encoded toxin. An attractive hypothesis that remains to be validated is that LlsB participates in the posttranslational modification of another molecule outside the LLS operon.

Our present results show that although LLS constitutive expression [strain F2365(pHELP::*llsA*)] can damage RBCs in a blood agar plate or in a phosphate-buffered saline (PBS) suspension, the concentration of LLS produced by the epidemic *L. monocytogenes* F2365 WT strain during *in vivo* infection once the intestine has been crossed has minimal toxicity or even no effect on eukaryotic cells. On the contrary, LLS is highly toxic for prokaryotic targets such as *L. lactis in vitro* and modulates the host microbiota *in vivo* (4). These data demonstrate that LLS is the first SLS-like virulence factor of a bacterial pathogen that only targets prokaryotic cells *in vivo*. It is very important to highlight that in our present experiments, LLS expression is detected in the stomach and the intestine of mice after intravenous bacterial inoculation. Indeed, it has been previously shown that *L. monocytogenes* can be discharged back to the gastrointestinal system from the gallbladder (23, 24). Our results suggest that modulation of the host microbiota by LLS takes place not only during oral animal infection but also upon intravenous infection: this fact should be now taken into account during *in vivo* animal experiments with relevant epidemic *L. monocytogenes* strains expressing the LLS operon.

## MATERIALS AND METHODS

### Bacterial strains and cell lines.

The epidemic lineage I *L. monocytogenes* strain F2365 of serotype 4b responsible for the 1985 California listeriosis outbreak (25) was used as parental strain (BUG3012; UIBC bacterial collection). The isogenic mutants and plasmids used in this study are listed in Table 1. Bacteria were grown in brain heart infusion (BHI) medium with shaking at 200 rpm in tubes at 37°C. *E. coli* cells were grown in LB broth. When required, antibiotics were added (chloramphenicol at 35 μg/ml for *E. coli* or 7 μg/ml for *L. monocytogenes*). The tissue culture cells used in this study were from the RAW264.7 (BALB/c mouse macrophage cells; ATCC TIB-71), HD11 (avian macrophage cell line [26]), and Caco-2 (ATCC HTB-37) lines. Cells were maintained in Dulbecco’s modified Eagle’s medium (DMEM) (Gibco) with 2 mM GlutaMAX (4 mM for RAW264.7 cells) supplemented with 10% (20% for Caco-2) (vol/vol) fetal calf serum (BioWest). Cells were grown at 37°C with 10% CO_2_.

**TABLE 1:** Bacterial strains used in this study^a^

BUG no.	Mutation/relevant genotype	Strain	Reference
3012	Wild type	*L. monocytogenes* 4b F2365	25
3651	PrfA*	*L. monocytogenes* 4b F2365	This study
3671	Δ*hly*	*L. monocytogenes* 4b F2365	This study
3781	Δ*llsA*	*L. monocytogenes* 4b F2365	4
3795	Δ*llsA*(pPl2::*llsA*)	*L. monocytogenes* 4b F2365	4
3817	pHELP::*llsA*	*L. monocytogenes* 4b F2365	4
3819	Δ*hly*(pHELP::*llsA*)	*L. monocytogenes* 4b F2365	This study
3672	Δ*llsB*	*L. monocytogenes* 4b F2365	4
3975	Δ*llsB*(pPl2::*llsB*)	*L. monocytogenes* 4b F2365	4
3783	Δ*llsA* PrfA*	*L. monocytogenes* 4b F2365	This study
3824	*inlB* corrected	*L. monocytogenes* 4b F2365	This study
4060	pAD-P*hly*-GFP	*L. monocytogenes* 4b F2365^InlB^	This study
4058	pAD-P*llsA*-GFP	*L. monocytogenes* 4b F2365^InlB^	This study
3763	LLS promoter fused to *lux* reporter system in pPL2 *lux*	*L. monocytogenes* 4b F2365	4
4048	pAD-P*llsA*-GFP	*E. coli*	This study
4052	pAD-P*hly*-GFP	*E. coli*	This study

a The strains shown are from the UIBC bacterial collection.

### Mutant construction.

To construct *L. monocytogenes* F2365 Δ*hly*, fragments containing 500 bp DNA flanking the open reading frames (ORFs) of *hly* were amplified by PCR and cloned into the suicide integrative vector pMAD as previously described (see Table S2 in the supplemental material) (27). The F2365 PrfA* mutant strain was designed inserting a point mutation (G145S) in PrfA, which rendered it constitutively active. The prfA*-A/prfA*-B and prfA*-C/prfA*-D (Table S2) oligonucleotides were designed to introduce a silent mutation in Cys144 (codon TGC→TGT) and a missense mutation in Gly145 changing it to Ser145 (codon GGT→TCT). The DNA fragment generated after splicing by overlap extension (SOEing) PCR was inserted into pMAD.

10.1128/mBio.00259-17.6 TABLE S2  Oligonucleotide primers used in this study for pMAD construction. Restriction sites are underlined. Download TABLE S2, DOC file, 0.1 MB. Copyright © 2017 Quereda et al. 2017 Quereda et al. This content is distributed under the terms of the Creative Commons Attribution 4.0 International license.

*L. monocytogenes* F2365 has a premature stop codon in *inlB* (codon no. 34 is TAA) (28). To facilitate *in vitro* cell infection and imaging, we generated an *L. monocytogenes* F2365 strain with a functional *inlB* (*L. monocytogenes* F2365^InlB^) by introducing a point mutation in codon 34 (TAA→CAA). The oligonucleotides InlB-new-A/InlB-new-B and InlB-new-C/InlB-new-D (Table S2) were used in SOEing PCR, and the PCR fragment was also cloned into pMAD.

To construct *L. monocytogenes* F2365 Δ*hly*(pHELP::*llsA*), we electroporated *L. monocytogenes* F2365 Δ*hly* with pMAD containing the promoter pHELP fused between two 500-nucleotide (nt) DNA fragments flanking the start codon of *llsA* (BUG3801 [4]). To construct the *L. monocytogenes* Δ*llsA* PrfA* strain, we electroporated *L. monocytogenes* F2365 PrfA*with pMAD-llsA from BUG3751 (4). Mutagenesis was performed by double recombination as described previously (27).

### Bacterial cocultures and electron microscopy.

Coculture assays of *L. monocytogenes* and *L. lactis* (Institut Pasteur Collection CIP 70.56T) were performed for 24 h in 6% O_2_ as previously described (4). At 3, 6, 9, and 24 h, part of the coculture was fixed for transmission electron microscopy, and another part of the culture was used to determine viable CFU on BHI agar plates. For biosafety reasons, bacterial strains or cocultures were inactivated by fixation with 2% glutaraldehyde (Sigma-Aldritch) in PHEM (PIPES-HEPES-EGTA-MgSO_4_⋅7H_2_O) buffer at pH 7.1. After inactivation, cells were washed with PHEM buffer and centrifuged. The pellet was resuspended in a small volume of PHEM buffer, and this suspension was taken up in capillary tubes (Wohlwendt Engineering) as described previously (29). The filled tube was separated by clamping into segments of less than 2 mm and placed into the 200-μm deep cavity of an aluminum planchette, type A (Wohlwendt Engineering) filled with 1-hexadecene. With the flat side of the complementary type B planchette, the filled planchette was closed and frozen with the HPM 010 (Abra fluid).

Freeze substitution was performed in anhydrous acetone containing 1% osmium tetroxide (Merck). 1-Hexadecene is insoluble at −90°C in dry acetone. To allow access of the substitution mix to the sample, small cracks were introduced under liquid nitrogen in the solid hexadecene by application of gentle pressure using a precooled fine-point forceps (Dumont). Substitution was carried out at −90°C for 24 h, at −30°C for 12 h, and at 0°C for 1 h each in a freeze substitution device FS8500 (RMC). Next, the samples were washed with dry acetone and embedded stepwise in Epon (29). After heat polymerization, thin sections were cut with a UC6 microtome (Leica Microsystems, Inc.). Sections were collected on 200-mesh Formvar-coated cupper grids and poststained with 4% aqueous uranyl acetate and Reynold’s lead citrate. Images were taken at 120 kV with a Tecnai G2 transmission electron microscope (FEI) equipped with a US4000 camera (Gatan, Inc.).

### Blood hemolysis assay on agar plates.

Hemolysis was assessed by streaking 10 µl of frozen bacterial cultures to isolate single colonies onto BHI agar plus 5% mouse (BALB/c) or human blood (French National Blood Service) and incubating the cultures for 24 h at 37°C.

### Live imaging of blood hemolysis.

Fresh whole human blood (French National Blood Service) was centrifuged (2,000 × *g*, 10 min, 4°C). Three components were obtained at this stage: (i) the upper phase, a clear solution of blood plasma; (ii) a middle thin layer of platelets and leukocytes; and (iii) at the bottom RBCs. RBCs were collected and washed twice in cold 1× Dulbecco’s PBS (DPBS; Gibco). An equivalent MOI of 40 bacteria was incubated with 2 ml of RBC suspension (≈10,000 RBCs) in 35-mm petri dishes (MatTek) at 37°C. Bacterial overnight cultures were directly added to the petri dishes during microscopy acquisition. Live-cell imaging was performed during 60 min on a Zeiss Axio Observer spinning-disk confocal microscope equipped with a 63×oil objective and driven by the MetaMorph software. Images were acquired every 5 s for 60 min. One hundred RBCs were counted for hemolysis for each of the strains tested during the duration of the experiment. Three independent experiments were performed.

### Whole-blood killing assays.

Fresh human whole blood (French National Blood Service) was diluted 1/5 into RPMI, and 96-well tissue culture plates were seeded with 100 μl of this suspension. The *L. monocytogenes* strains were grown overnight in BHI, washed in PBS, and diluted in RPMI medium. A total of 5 × 10^4^ bacteria were added per well. The mixture of bacteria and blood was incubated at 37°C for 2, 4, and 24 h. The number of *L. monocytogenes* survivors was determined by serial dilution and colony counting on BHI agar plates. The experiments were repeated with blood from three independent human donors. Six technical replicates per bacterial strain and per blood donor were performed using independently derived clones of each of the strains. Statistical analyses were performed using the Student’s *t* test.

### Cell infection.

Prior to infection, 96-well tissue culture plates were seeded with cells to attain 80% confluence on the day of infection. Overnight cultures of bacterial strains were washed three times in PBS and resuspended in infection medium (1% fetal bovine serum [FBS]) at an MOI of 2 (RAW264.7 and HD11) or 5 (Caco-2). Cells were centrifuged for 1 min at 1,000 rpm to synchronize infection. The cells were then incubated with the bacteria for 1 h at 37°C. Following this incubation, the cells were washed, and extracellular bacteria were neutralized by adding complete medium containing 40 µg/ml of gentamicin. At 2, 6, or 24 h postinfection, cells were washed with PBS and finally lysed in distilled water containing 0.1% Triton X-100. The number of viable intracellular *L. monocytogenes* cells was calculated by serial dilution and colony counting on BHI agar plates. These experiments employed 6 technical replicates per bacterial strain and were repeated three times with independent clones of each of the strains. Statistical analyses were conducted by using the Student’s *t* test.

### Cytotoxicity LDH release assays and cytokine measurements.

RAW264.7 and Caco-2 cells were infected as indicated in the previous paragraph for 24 h (washed after 1 h of infection and with 40 µg/ml gentamicin added to kill extracellular bacteria) where the supernatant was recovered and filtered. LDH levels were assayed with the kit LDH BR (Linear Chemicals) according to the kit instructions. Supernatants from RAW264.7 cells were also used to measure cytokine levels by using the cytokine array kit (Proteome Profiler mouse XL cytokine array; Becton, Dickinson) according to the manufacturer’s instructions.

### Vacuolar escape.

RAW264.7 cells were infected as indicated in the previous paragraphs for 6 h (washed after 1 h of infection and with 40 µg/ml gentamicin added to kill extracellular bacteria) and fixed with a paraformaldehyde (PFA) solution (4% in PBS) for 15 min at room temperature. Extracellular *L. monocytogenes* cells were labeled with a primary polyclonal goat anti-*Listeria* serum and with a secondary chicken anti-goat Alexa 647. Next, cells were permeabilized using 0.1% Triton X-100 for 4 min at room temperature, and total *L. monocytogenes* cells were labeled with the same primary antibody and a secondary chicken anti-goat Alexa 488 antibody. Actin was labeled by using phalloidin conjugated to Alexa 546. Nuclei were stained with Hoechst 33342 (dilution, 1/1,000). Samples were then rinsed four times in PBS and examined with a Zeiss Axiovert 135 epifluorescence microscope (Carl Zeiss, Inc.) associated with a charge-coupled device (CCD) camera. Images were obtained with a ×63 oil immersion objective and processed with MetaMorph software (Universal Imaging). Cytosolic bacteria were considered those stained with the Alexa 546 and Alexa 488 antibody but lacking Alexa 647 antibody labeling. Approximately 100 cells were counted in 3 representative fields to estimate the number of cytosolic *L. monocytogenes* cells.

### Evaluation of *llsA* promoter expression with a GFP and a luciferase reporter system.

A transcriptional fusion of the *llsA* promoter was created by cloning 500 bp upstream of the ATG of the respective gene with the gene encoding GFP-mut2 (30) (generating P*llsA*-GFP). This construct was cloned into SalI/SmaI-digested pAD vector (generating pAD-P*llsA*-GFP). Gene synthesis to construct P*llsA*-GFP was produced by Genecust (Luxembourg). pAD-P*llsA*-GFP was electroporated into *L. monocytogenes* F2365^InlB^. *hly* was used as a control gene, the expression of which is upregulated during infection of eukaryotic cells. The transcriptional fusion of the promoter of *hly* to GFP-mut 2 (pAD-P*hly*-GFP) was also cloned into *L. monocytogenes* F2365^InlB^.

For epifluorescence analysis of promoter activity, RAW264.7 and Caco-2 cells were infected for 6 h (washed after 1 h of infection and with 40 µg/ml gentamicin added) fixed with a PFA solution (4% in PBS) for 15 min at room temperature, and permeabilized (0.1% Triton X-100 for 3 min in PBS). Samples were then rinsed four times in PBS, incubated with Hoechst and phalloidin conjugated to Alexa 546 for 30 min at room temperature, and rinsed four times in PBS. Samples were examined with a Zeiss Axiovert 135 epifluorescence microscope (Carl Zeiss, Inc.) associated with a charge-coupled device (CCD) camera. Images were obtained with a ×63 oil immersion objective and processed with MetaMorph software (Universal Imaging).

For *in vivo* bioluminescence experiments, 8-week-old female BALB/c mice were infected by intravenous inoculation with 10^4^ *L. monocytogenes* F2365^*llsA*::*lux*^ (BUG3763) cells grown in BHI broth to an optical density (OD) of 1.0 at 37°C. Bioluminescence imaging was accomplished using an IVIS Spectrum *in vivo* imaging system (Perkin Elmer) with a 5-min exposure time. Mice were anesthetized with isoflurane. For CFU determinations, whole luminal contents from stomach, small intestine, cecum, and colon, as well as tissues from stomach, small intestine, cecum and colon, liver, and spleen, were obtained, homogenized, and serially diluted and plated on Oxford agar plates (Oxoid). In order to determine the CFU numbers in the tissues of the gastrointestinal system, the tissues were washed three times in DMEM, incubated for 2 h in DMEM supplemented with 40 μg/ml gentamicin, and finally washed three times in DMEM.

### Mouse infections.

Six- to 8-week-old female BALB/c mice (Charles River, Inc., France) were injected intravenously with 10^4^ CFU of the indicated strain. Mice were sacrificed at 48 h after infection (four mice in each group), and livers and spleens were removed. Half of the organ was used to assess bacterial load, and the other half was used for histological analysis. To assess bacterial load, organs were homogenized and serially diluted. Dilutions were plated onto BHI plates and grown during 24 h at 37°C. Colonies were counted to assess bacterial load per organ. RBC and hemoglobin counts were determined using a Horiba scil Vet abc Plus veterinary hematology blood analyzer. Two independent experiments were carried out. Statistically significant differences were evaluated by the Mann-Whitney test. Pearson’s correlation coefficients were computed to measure correlations between blood parameters and *L. monocytogenes* CFU in blood.

### Histological analysis.

Liver and spleen tissue sections from mice intravenously infected with the F2365 WT, Δ*llsA*, and Δ*llsA* complemented strains sacrificed at 48 h were fixed in 10% neutral buffered formalin and routinely processed for the histopathological analysis. Four-micrometer sections per organ were stained with hematoxylin and eosin (H&E). The number of necrotic foci and the main cell type infiltrating necrotic areas were recorded. All of the slides were internally coded and analyzed blind. Statistically significant differences were evaluated by the Mann-Whitney test.

### Ethics statement.

This study was carried out in strict accordance with the French national and European laws and conformed to the Council Directive on the approximation of laws, regulations, and administrative provisions of the Member States regarding the protection of animals used for experimental and other scientific purposes (86/609/Eec). Experiments that relied on laboratory animals were performed in strict accordance with the Institut Pasteur’s regulations for animal care and use protocol, which was approved by the Animal Experiment Committee of the Institut Pasteur (approval no. 03-49). All human blood samples were anonymized and collected from the French National Blood Service under IRB approval no. HS2008-3470.
