# Supplementary material for: Listeriolysin S Is a Streptolysin S-Like Virulence Factor That Targets Exclusively Prokaryotic Cells In Vivo
Source: mBio. 2017 Apr 4;8(2):e00259-17. doi: 10.1128/mBio.00259-17 (PMC5380841; doi:10.1128/mBio.00259-17)
Supplement: TABLE S1 [file mbo002173256st1.doc]

**S1 Table.**

|  | **CFU/Blood ml** | **RBC / ml** | **Hemoglobin g/dl** |
| --- | --- | --- | --- |
| **WT1** | 0 | 10234607.51 | 20.83823 |
| **WT2** | 400 | 8803244.681 | 18.58085 |
| **WT3** | 500 | 9935263.158 | 20.25526 |
| **WT4** | 50 | 8151818.182 | 17.03864 |
| **WT5** | 11250 | 8381148.649 | 17.3 |
| **Δ*llsA*1** | 50 | 7766566.265 | 16.10843 |
| **Δ*llsA*2** | 50 | 8382758.621 | 18.05517 |
| **Δ*llsA*3** | 5100 | 6549565.217 | 14.85217 |
| **Δ*llsA*4** | 6550 | 8518604.651 | 16.77907 |
| **Δ*llsA*5** | 300 | 8740869.565 | 18.01739 |
| **Δ*llsA*+*llsA*1** | 50 | 9662516.556 | 19.81457 |
| **Δ*llsA*+*llsA*2** | 50 | 7265892.857 | 15.4125 |
| **Δ*llsA*+*llsA*3** | 2100 | 8405434.783 | 16.47011 |
| **Δ*llsA*+*llsA*4** | 3000 | 9832920.792 | 19.32871 |
| **Δ*llsA*+*llsA*5** | 50 | 9007935.223 | 18.94089 |
