# Supplementary material for: Listeriolysin S Is a Streptolysin S-Like Virulence Factor That Targets Exclusively Prokaryotic Cells In Vivo
Source: mBio. 2017 Apr 4;8(2):e00259-17. doi: 10.1128/mBio.00259-17 (PMC5380841; doi:10.1128/mBio.00259-17)
Supplement: TABLE S2 [file mbo002173256st2.doc]

**S2 Table.**

| Name | Sequence 5’-3’ | Purpose of use |
| --- | --- | --- |
| prfa*-a | ctgatcggatccgctaacaattgttgttactgcc | Amplification of ~500 bp fragment in flanking region of *prfA* |
| prfa*-b | gattaaaagttgagaacaaatagagcc | Amplification of ~500 bp fragment in flanking region of *prfA* |
| prfa*-c | ctatttgttctcaacttttaatcctgac | Amplification of ~500 bp fragment in flanking region of *prfA* |
| prfa*-d | gagtcacccgggcaatcacacttgctgctaaag | Amplification of ~500 bp fragment in flanking region of *prfA* |
| prfa*-x | atatctccgagcaaccatcg | Verification of the *prfA** mutation (mapping in reading frame) |
| prfa*-y | agcgccgattgctattattg | Verification of the *prfA** mutation (mapping in reading frame) |
| hly-a | aatgacccggggttttgctcgtcttttaaacgcat | Amplification of ~500 bp fragment in flanking region of *hly* |
| hly-b | gtgttaagcggttttattcttaatttttgggtttcactctccttctacat | Amplification of ~500 bp fragment in flanking region of *hly* |
| hly-c | aaaaattaagaataaaaccgcttaacac | Amplification of ~500 bp fragment in flanking region of *hly* |
| hly-d | tcgaggtcgactcatcattatcagtcaagtaaccat | Amplification of ~500 bp fragment in flanking region of *hly* |
| hly-x | tttgattagtaatcctaagctgcc | Verification of the Δ*hly* mutation (mapping in reading frame) |
| hly-y | tagggattttattgctcgtgtca | Verification of the Δ*hly* mutation (mapping in reading frame) |
| InlB-new-A | ctgatcggatccgctgtacgctcaattcacga | Amplification of ~500 bp fragment in flanking region of codon 34 of *inlB* |
| InlB-new-B | tctccgcttgtactttcgccc | Amplification of ~500 bp fragment in flanking region of codon 34 of *inlB* |
| InlB-new-C | ggcgaaagtacaagcggagac | Amplification of ~500 bp fragment in flanking region of codon 34 of *inlB* |
| InlB-new-D | gagtcagaattctcctgctaacgctcttaaatcgc | Amplification of ~500 bp fragment in flanking region of codon 34 of *inlB* |
| F2365-InlB-testF | accaaaaacgccaacaaaag | Verification of the *inlB* mutation (mapping in reading frame) |
| F2365-InlB-testR | aacaagcgacccatcaatgt | Verification of the *inlB* mutation (mapping in reading frame) |
